# Screening of Specific and Common Pathways in Breast Cancer Cell Lines MCF-7 and MDA-MB-231 Treated with Chlorophyllides Composites

**DOI:** 10.3390/molecules27123950

**Published:** 2022-06-20

**Authors:** Keng-Shiang Huang, Yi-Ting Wang, Omkar Byadgi, Ting-Yu Huang, Mi-Hsueh Tai, Jei-Fu Shaw, Chih-Hui Yang

**Affiliations:** 1 The School of Chinese Medicine for Post-Baccalaureate, I-Shou University, No. 8, Yida Rd., Jiaosu Village Yanchao District, Kaohsiung City 82445, Taiwan; huangks@isu.edu.tw; 2 Department of Biological Science and Technology, I-Shou University, No. 8, Yida Rd., Jiaosu Village Yanchao District, Kaohsiung City 82445, Taiwan; teengina1220@isu.edu.tw (Y.-T.W.); z09170707@isu.edu.tw (T.-Y.H.); mihsueh@isu.edu.tw (M.-H.T.); 3 International College, International Program in Ornamental Fish Technology and Aquatic Animal Health, National Pingtung University of Science and Technology, No. 1, Shuefu Road, Neipu, Pingtung 91201, Taiwan; omkarcof1@gmail.com; 4 Pharmacy Department, E-Da Hospital, No. 1, Yida Rd., Jiaosu Village Yanchao District, Kaohsiung City 82445, Taiwan; 5 Taiwan Instrument Research Institute, National Applied Research Laboratories, Taipei City 106214, Taiwan

**Keywords:** chlorophyllides, microarray-based detection, breast cancer, MCF-7, MDA-MB-231

## Abstract

Our previous findings have shown that the chlorophyllides composites have anticancer activities to breast cancer cell lines (MCF-7 and MDA-MB-231). In the present study, microarray gene expression profiling was utilized to investigate the chlorophyllides anticancer mechanism on the breast cancer cells lines. Results showed that chlorophyllides composites induced upregulation of 43 and 56 differentially expressed genes (DEG) in MCF-7 and MDA-MB-231 cells, respectively. In both cell lines, chlorophyllides composites modulated the expression of annexin A4 (ANXA4), chemokine C-C motif receptor 1 (CCR1), stromal interaction molecule 2 (STIM2), ethanolamine kinase 1 (ETNK1) and member of RAS oncogene family (RAP2B). Further, the KEGG annotation revealed that chlorophyllides composites modulated DEGs that are associated with the endocrine system in MCF-7 cells and with the nervous system in MDA-MB-231 cells, respectively. The expression levels of 9 genes were validated by quantitative reverse transcription PCR (RT-qPCR). The expression of CCR1, STIM2, ETNK1, MAGl1 and TOP2A were upregulated in both chlorophyllides composites treated-MCF-7 and MDA-MB-231 cells. The different expression of NLRC5, SLC7A7 and PKN1 provided valuable information for future investigation and development of novel cancer therapy.

## 1. Introduction

Breast cancer is the second most likely cause of cancer-related mortality in women [1,2,3]. It is evident that molecular alterations or epigenetic modifications in cancerous cells leads to the formation of the malignancies [4]. Clinical classifications of breast cancers were based on the status of estrogen receptor (ER), progesterone receptor (PR) and human epidermal growth receptor 2 (HER2) [5]. Generally, MCF-7 includes ER-positive and PR-positive breast cancer cell lines [6]. MDA-MB-231 cells are triple-negative breast cancer (TNBC), which are negative for ER, PR and HER2. Morphologically, MCF-7 and MDA-MB-231 cells are both epithelial cells that are derived from mammary gland carcinoma cells. Histologically, MCF-7 is a luminal type of breast cancer, while MDA-MB-231 is a basal type. MCF-7 cells were effectively treated with drugs, such as tamoxifen paclitaxel, docetaxel or doxorubicin. MDA-MB-231 cells (TNBC) are found to be aggressive, prone to relapse, have high metastasis rate and poor prognosis and are insensitive to treatment [7,8,9,10,11].

Current treatment for breast cancer is a multi-strategy approach, including chemotherapy, surgery, radiotherapy and endocrine therapy [5,12,13]. Treatment for breast cancer (stages I-III) includes surgery and radiation therapy, with chemotherapy or other drug therapies administered before (neoadjuvant) or after (adjuvant) surgery. Chemotherapy is used mainly for downstaging, shrinking of tumors or to determine the response to therapy during early-stage breast cancer and to locally advanced breast cancer [14]. Cancer can be removed by surgery, including lumpectomy and then by whole-breast irradiation or mastectomy [15]. Whole breast irradiation is relatively acceptable, but it has inevitable acute and delayed toxic effects [16,17]. Systemic chemotherapy using cytotoxic agents or endocrine therapy are the major treatment strategy for metastatic breast cancer [12]. For example, the commonly used chemotherapy drugs—paclitaxel was diterpene (C20) formed through the condensation of four isoprene molecules [18,19]. Generally, paclitaxel was applied as a first-line (adjuvant) treatment of node-positive breast cancer [20]. For the metastatic breast cancer which has failed after combination of chemotherapy or relapse of adjuvant chemotherapy (within 6 months), paclitaxel was app clied as a second line agent in breast cancer. The cytotoxic effects of paclitaxel are targeting of p53, inducing cellular apoptosis and mitotic arrest [21]. Natural compounds that are derived from plants have interesting biological activities, including antimicrobial, anti-oxidation, anti-inflammatory and cytotoxic effects [22]. The diverse range of compounds that showed potential inhibitory effects against oncogenic transcription factors in breast cancer were surveyed and reviewed, namely edomin, triterpenoids, parthenolide, vincristine, irinotecan, green tea polyphenol and several others [5,12]. 

Although there are several technologies for the identification of differentially expressed genes after treatment, microarray is a precise and thorough tool [23]. The advantages of microarray for gene detection are rapid, high accuracy and comprehensive detection [24,25]. Microarray technology has been used to study gene expression during the oncogenesis, metastasis or drug resistance during cancers [26,27,28]. Similarly, it can also be applied to the diagnosis, classification, prognosis and screening of drug targets involved in the treatment of breast cancer [29,30,31]. For example, microarray could be applied in cell lines with different stages of metastasis to obtain metastasis-related genes [32,33]. Further, to compare the differential expressions between different subtypes of breast cancers (e.g., MCF-7 and MDA-MB-231 cells), a large number of tumor-specific markers could be screened. Therefore, advances in microarray has made it possible and accelerated the overall study on the differentially expressed genes in breast cancer. 

MDA-MB-231 cells are insensitive to conventional breast cancer treatments (e.g., chemotherapy, endocrine therapy or targeted therapy) due to drug resistance and metastasis [34,35,36]. The differentially expressed genes (DEGs) may be involved in signaling pathways, such as apoptosis, cell cycle and cell growth and thus have been common therapy targets in drug-resistant breast cancers. Although drugs that target important factors in signaling pathways may activate another or inhibit to resist drug effect, therapies targeting those factors have offered promising results for preventing breast cancer. Our previous studies have demonstrated that chlorophyllide composites have potential in the treatment of MDA-MB-231 cells [37,38]. However, the underlying molecular mechanisms involved have yet to be fully elucidated. Understanding the mechanisms that drive drug resistance is important to the development of novel treatment and to increase the surveillance in patients. Therefore, the main aim of this study is to characterize the effects of chlorophyllides on MCF-7 and MDA-MB-231 cells through microarray gene expression profiling. We compared the gene expression profiles among chlorophyllides composites-treated MCF-7 and MDA-MB-231 cells to exhibit the cancer-related or drug resistance-related molecular mechanism and frame a possible strategy to develop as a target of botanical drugs. 

## 2. Materials and Methods

### 2.1. Materials

Ethanol and *n*-hexane were purchased from Seedchem Company Pty., Ltd. (Melbourne, Australia). 3-(4,5-dimethylthiazol-2-yl)-2,5-diphenyl tetrazolium bromide (MTT), potassium hydroxide, sodium phosphate, Triton™ X-100 and chlorophyll a/b standards were purchased from Sigma-Aldrich, Inc. (St. Louis, MO, USA). Sweet potato leaves were purchased from a local market in Kaohsiung, Taiwan. Human breast cancer cell lines (MCF-7 and MDA-MB-231) were purchased from the Bioresource Collection and Research Center (Food Industry Research and Development Institute, Taiwan). Dulbecco’s modified Eagle’s medium (DMEM) and fetal bovine serum (FBS) were obtained from Invitrogen (Carlsbad, CA, USA). TRIzol^®^ reagent was purchased from Invitrogen Corp. (Carlsbad, CA, USA). 

### 2.2. Chlorophyll Extraction and Measurement

Chlorophyll was obtained as described from the laboratory of Prof. Shaw [37]. Fresh leaf samples were washed with water and blotted. Ten grams of fresh, clean leaves were weighted and ground into powders using a mortar and pestle, with liquid nitrogen (50 mL) in the dark. Chlorophyll was extracted by immersing 1 g of leaf powder in 125 mL of ethanol. After 48 h, ethanol extract was centrifuged at 1500× *g* for 5 min. The chlorophyll from ethanol extracts were then sequentially extracted using *n*-hexane. After 48 h, the double extract of chlorophylls was centrifuged at 1500× *g* for 5 min, and purified chlorophylls from ethanol-hexane extracts were obtained. The concentrations of chlorophyll *a*/*b* were measured by UV-Vis spectrophotometer (BioTek Instruments, Inc., Winooski, VT, USA) for the absorbance at 649 and 665 nm, which are the major absorption peaks for chlorophyll *a* and *b*, respectively. The chlorophyll *a* and *b* contents of the leaves were calculated using previously devised equations [39].

### 2.3. Preparation of Chlorophyllides Composites by Using Chlorophyllase

Chlorophyllase was obtained as described from the laboratory of Prof. Shaw [40]. The reaction mixture contained 0.5 mg of recombinant chlorophyllase, 650 µL of the reaction buffer (100 mM sodium phosphate (pH 7.4) and 0.24% Triton X-100) and 0.1 mL of chlorophyll extracts from the sweet potato leaves (100 mM). The reaction mixture was incubated at 37 °C for 30 min in a shaking water bath, then the enzymatic reaction was stopped by adding 1 mL of 10 mM KOH. The reaction mixture was then vortexed vigorously and centrifuged at 4000 rpm for 10 min to collect chlorophyllides composites. Chlorophyllides composites were then concentrated, and the solvents were removed by evaporation under reduced pressure at 40 °C on a rotary evaporator (IKA-Werke, Germany). The concentrated composites were processed by lyophilization, weighed and stored at −80 °C for further experiments. All compounds were found to be >95% pure by HPLC analysis (Figure S1). 

### 2.4. Total RNA Preparation for Sequencing

Breast cancer cell lines (MCF-7 and MDA-MB-231), cultured in DMEM supplemented with 10% FBS and maintained at 37 °C under a humidified atmosphere of 5% CO_2_ with 5 × 10^4^ cells/well, were treated with 100 μg/mL of prepared chlorophyllides composites [37,38] or DMSO (vehicle control). Cells were collected at 24 h after treatment and shipped using dry ice to Welgene Biotech. Co., Ltd., Taipei, Taiwan. 

Total RNA was extracted using TRIzol^®^ reagent according to the manufacturer’s instructions [41]. The RNA quality was confirmed using the ratios A260/280 and A260/230 (Thermo fisher scientific Inc., Waltham, MA, USA). RNA concentration and integrity were analyzed by Bioanalyzer 2100 total RNA Nano series II chip (Agilent, Santa Clara, CA, USA). 

### 2.5. Preparation of cDNA Library and Sequencing

RNA integrity was assessed using the RNA Nano 6000 Assay Kit on the Bioanalyzer 2100 system (Agilent Technologies, Santa Clara, CA, USA). 0.2 μg of total RNA was amplified by a Low Input Quick-Amp Labeling kit (Agilent Technologies, Santa Clara, CA, USA) and labeled with Cy3 (CyDye, Agilent Technologies, Santa Clara, CA, USA) during the in vitro transcription process. 0.6 μg of Cy3-labled cRNA was fragmented to an average size of about 50–100 nucleotides by incubation with fragmentation buffer at 60 °C for 30 min [42]. 

### 2.6. Microarray Gene Expression Profiling

Microarray profiling was performed using Agilent SurePrint Microarray (Agilent Technologies, Santa Clara, CA, USA) at 65 °C for 17 h After washing and drying by nitrogen gun blowing, microarrays were scanned with an Agilent microarray scanner (Agilent Technologies, Santa Clara, CA, USA) at 535 nm for Cy3. Scanned images were analyzed by Feature Extraction 10.7.3.1 software (Agilent Technologies, Santa Clara, CA, USA), an image analysis and normalization software was used to quantify signal and background intensity for each feature. Raw signal data was normalized by quantile normalization for differential expressed genes discovery. For functional assay, enrichment tests for gene ontology (GO) and KEGG pathway were performed for DEGs by clusterProfiler.

### 2.7. Quantitative Reverse Transcription PCR (RT-qPCR)

Validation of RNA-Seq data was performed by RT-qPCR. DNase I-treated total RNA from chlorophyllides composites-treated MCF-7 and MDA-MB-231 cells was subjected to cDNA synthesis using iScript™ cDNA synthesis kits (Bio-Rad, Hercules, CA, USA). PCR primers were designed based on the transcriptome sequences of annexin A4 (ANXA4), chemokine C-C motif receptor 1 (CCR1), stromal interaction molecule 2 (STIM2), ethanolamine kinase 1 (ETNK1) and member of RAS oncogene family (RAP2B) using Primer 2 Plus software (Table 1). GAPDH served as the internal control, and RT-qPCR was performed using iQSYBR Green Supermix (Bio-Rad Laboratories, Hercules, CA, USA), and each sample was run in triplicate. The thermal gradient feature (CFX96, Bio-Rad Laboratories) was used to determine the optimal annealing temperature for all primers. The real-time PCR program used was 95 °C for 3 min, followed by 40 cycles of 95 °C for 15 s, 58 °C for 15 s, and 72 °C for 35 s. Dissociation and melting curves of amplification products were performed and results were analyzed using the CFX Manager Software package (Bio-Rad Laboratories). The 2^′∆∆Ct^ method was chosen as the calculation method [43]. The difference in the cycle threshold (Ct) value of the target gene and its housekeeping gene (GAPDH), called ∆Ct, was calculated using the following equation: ∆∆Ct = (∆Ct of chlorophyllides composites treatment or vehicle control group for the target gene at each time point) − (∆Ct of the initial control).

### 2.8. Statistical Analysis

Statistical comparisons were carried out by independent *t*-test using SPSS statistical software version 22.0 (SPSS Inc., Chicago, IL, USA). Values are shown as mean ± standard deviation. The acceptable level for statistical significance was *p* < 0.05.

## 3. Results and Discussion

### 3.1. DEG Analysis in MCF-7 and MDA-MB-231 Cells

**Figure 1:**
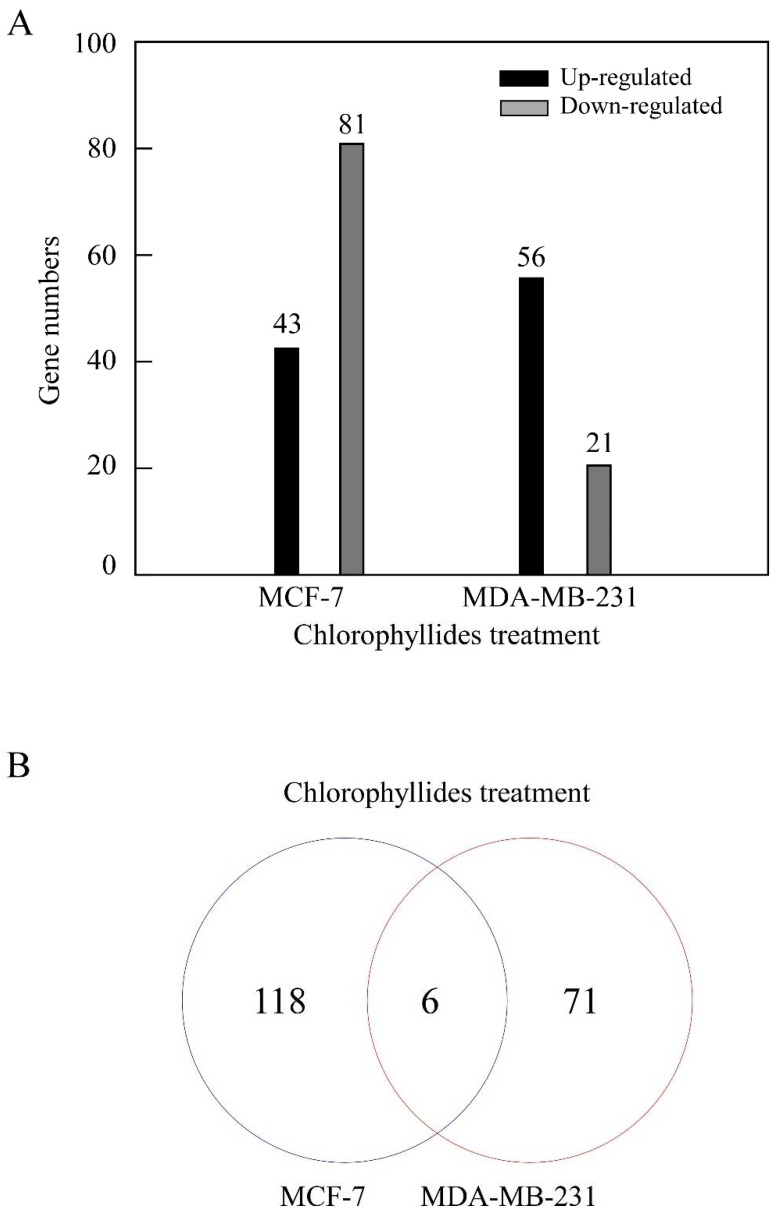
Comparisons of differentially expressed genes (DEGs) from MCF-7 and MDA-MD-231. (**A**) Upregulation and downregulation of DEGs in MCF-7 and MDA-MD-231 cells with chlorophyllides treatments. (**B**) Venn diagram of the overlapped DEGs between chlorophyllides-treated MCF-7 and MDA-MB-231 cells. The MCF-7 and MDA-MB-231 cells shared six genes of DEGs.

MCF-7 cells and MDA-MB-231 cell were subjected to chlorophyllides composites treatment, and the gene expression levels were compared. The data indicated that a total of 124 and 77 DEGs were specifically identified in MCF-7 and MDA-MB-231 cells, respectively (Figure 1A). These included 43 upregulated and 81 downregulated genes in MCF-7 and 56 upregulated and 21 downregulated genes in MDA-MB-231 cells (≥2-fold change (FC), *p* < 0.05). To analyze the common and specific DEGs in the two cells, a Venn diagram was also performed. There were 118 specific DEGs for the chlorophyllides composites-treated MCF-7 cells and 71 specific DEGs for the chlorophyllides composites-treated MDA-MB-231 cells. We first identified the 6 overlapped genes were found between chlorophyllides composites-treated MCF-7 and MDA-MB-231 cells (Figure 1B). The results revealed that the significant differences in the chlorophyllides composites-treated MCF-7 and MDA-MB-231 cells as compared to their parental cells. Therefore, chlorophyllides composites targeted and affected the expression of DEGs, indicating that chlorophyllides composites specifically inhibited the proliferation of MCF-7 and MDA-MB-231 cells. This finding is consistent with our previous results [38]. Furthermore, a higher number of DEGs was found in MCF-7, indicating that chlorophyllides composites were more efficient in MCF-7 cells. 

### 3.2. GO Annotation of Differential Expessed Genes

Comparison of gene expression levels between the MCF-7 cells, subjected to chlorophyllides composites and control cells, a total of 1383 GO terms were enriched. 29, 39 and 26 genes were clustered into three categories, namely biological process (BP), cellular component (CC) and molecular function (MF). The most enriched groups with 4 genes in MF were purine nucleoside binding and nucleoside binding. Eight groups in BP were enriched, including mesenchyme development, negative regulation of catabolic process, maintenance of location, negative regulation of cell migration, negative regulation of cell motility, negative regulation of cellular component movement, negative regulation of locomotion and muscle system process. The most enriched groups with 4 genes in CC were cell leading edge and synaptic membrane. 

Comparison of gene expression levels between chlorophyllides composites-treated MDA-MB-231 cells and control cells revealed that a total of 1051 GO terms were enriched. Twenty-five, 27 and 16 genes were clustered into BP, CC, and MF. Fourteen groups in MF were enriched, such as enzyme activity, cytokine activity or receptor ligand activity. The most enriched groups with 2 genes in BP were organelle fission and response to nutrient levels. The most enriched groups in CC were tertiary granule, ficolin-1-rich granule, secretory granule membrane, postsynaptic density, asymmetric synapse, postsynaptic specialization, neuron to neuron synapse, nuclear chromatin and microtubule.

**Figure 2:**
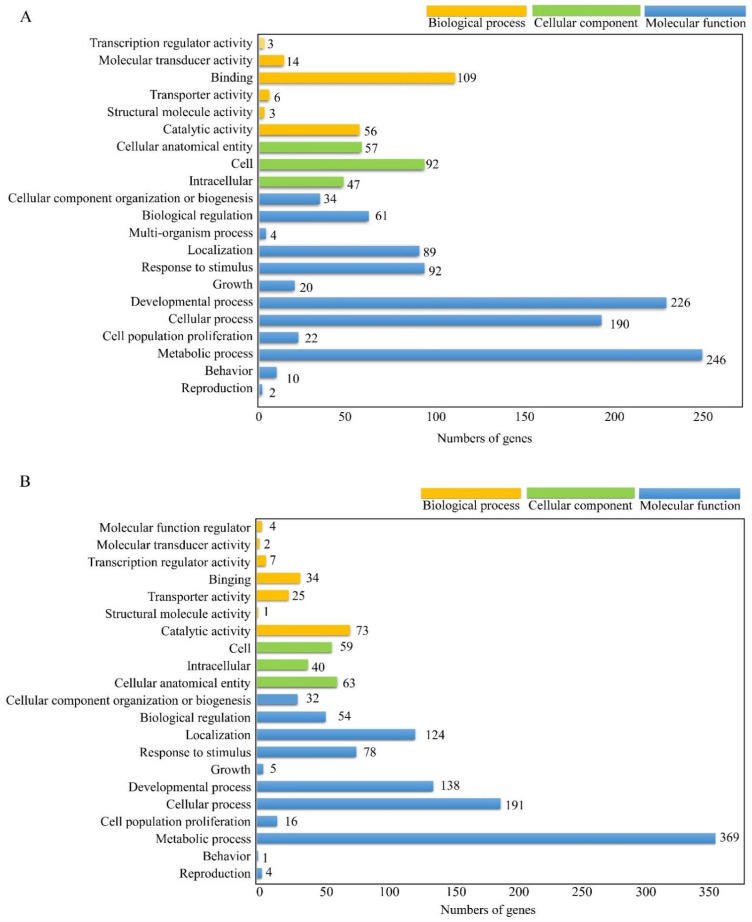
Functional distribution of GO annotation extracted from chlorophyllides composites treatments. (**A**) MCF-7 cells with chlorophyllides composites treatments. (**B**) MDA-MB-231 cells with chlorophyllides composites treatments. The results of GO enrichment analysis of DEGs were classified into three categories: molecular functions, cellular component and biological process. The *y*-axis is gene functional classification of GO, while *x*-axis is the corresponding number of genes.

To investigate the functional roles of the DEGs, specific DEGs with chlorophyllides composites treatment were mainly functionally annotated and assigned to GO terms (Figure 2). In MCF-7 cells, specific DEGs with chlorophyllides composites treatment were assigned to 958 GO terms based on sequence homology, and a total of 21 functional groups were clustered into BP, CC and MF (Figure 2A). The unigene sequences from MF were clustered into 6 different classifications. The largest subcategory within MF was “binding”, followed by “catalytic activity”. Twenty genes and 11 genes were annotated for binding and catalytic activity out of total MF. In the CC, sequences were distributed into 3 classifications. The most represented subcategories were “cell”, followed by “cellular anatomical entity”. “Metabolic process” with 246 GO annotations was the most represented among 12 subcategories within the BP, followed by “development process” for 226 GO annotations.

In MDA-MB-231 cells, specific DEGs were annotated and shown in Figure 2B. Those DEGs with chlorophyllides composites treatment were ascribed to 590 GO terms and divided into 21 functional groups (Figure 2B). The unigene sequences from MF were clustered into 7 different classifications. The largest subcategory within MF was “catalytic activity”, followed by “binding”. In the CC, sequences were distributed into 3 classifications. The most represented subcategories were “cellular anatomical entity”, followed by “cell”. “Metabolic process” with 369 GO annotations was the most represented among 11 subcategories within the BP, followed by “cellular process” for 191 GO annotations.

It has been reported that ursolic acid, quercetin, curcumin and kaempferol are potential anti-cancer compounds in the treatment of breast cancer [44,45,46,47]. Guo et al. evaluated the anti-cancer mechanism of ursolic acid by microarray [48]. They indicated that the effects of ursolic acid were by inhibition of nuclear factor kappa-B kinase (IKK)/nuclear factor kappa-B (NF-κB) and serine/threonine kinase protein (RAF)/ERK pathways in MCF-7 cells [48]. Bachmeier et al. reported that curcumin inhibited the phosphorylation of the IKK in breast cancer cells [49]. In the present study, our results demonstrated that the group-biological process was enriched with chlorophyllides composites treatment, indicating that chlorophyllides composites treatment may affect the metabolism, cell communication, or development. Therefore, the functional annotations of unigenes according to the GO database provide ample numbers of candidate genes and valuable information of the biological activity of chlorophyllides composites treatment in MCF-7 and MDA-MB-231 cells. 

### 3.3. KEGG Pathway Analysis of DEGs 

**Figure 3:**
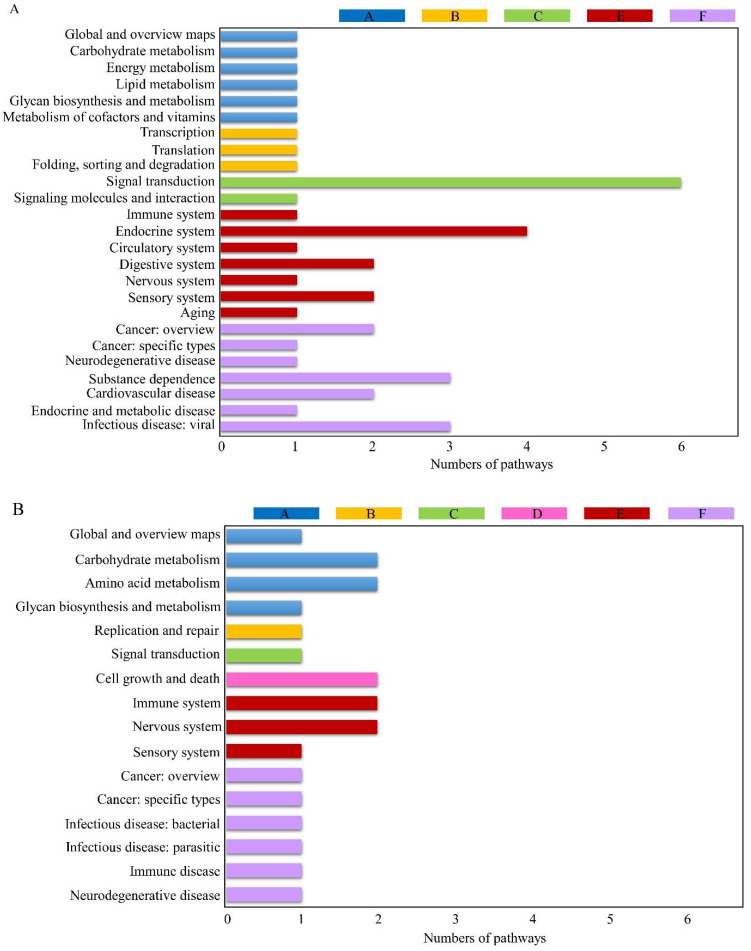
KEGG (Kyoto Encyclopedia of Genes and Genomes) assembled unigenes from chlorophyllides composites treatments. (**A**) MCF-7 cells; (**B**) MDA-MB-231 cells. The results were classified into six categories: A. metabolism; B. genetic information processing; C. environmental information processing; D. cellular processes; E. organismal systems; and F. human diseases. The *y*-axis is classification of KEGG, and the *x*-axis is the corresponding numbers of pathways. *p* < 0.5.

Overall, specific DEGs from MCF-7 with chlorophyllides composites treatment had significant matches, which were allocated to 41 KEGG pathways classified into 6 main categories, namely metabolism, genetic information processing, environmental information processing, cellular processes, organismal systems and human diseases (Figure 3A). The highest number of genes (13 gene) categorized from KEGG analysis related to human diseases with sub-groups from viral infectious disease (3 genes), substance dependence (3 genes), cardiovascular disease (2 genes), cancer (2 genes in overview and 1 gene in specific types), neurodegenerative disease (1 gene) and endocrine and metabolic disease (1 gene). Ten genes were related to organismal systems, where the majority of the genes were categorized as endocrine system (4 genes), followed by digestive system (2 genes), sensory system (2 genes), immune system (1 gene), circulatory system (1 gene), nervous system (1 gene) and aging (1 gene). Seven genes related to environmental information processing were categorized as signal transduction (6 genes) and signaling molecules and interactions (1 gene). Six and 3 genes were related to metabolism and genetic information processing, respectively. However, no genes were categorized into cellular processes. 

Moreover, DEGs from MDA-MB-231 cells with chlorophyllides composites treatment were allocated to 22 KEGG pathways (Figure 3B). The highest number of genes categorized from KEGG analysis related to organismal systems with sub-groups from the nervous or immune systems (2 genes). Seven pathways with 3 genes (RGS9, IL1B and SPl1) were related to human disease. It is interesting that IL1B was categorized into 6 pathways, including of hsa05010, hsa05321, hsa05146 and hsa05152. Three genes were allotted to metabolism with subgroups of global and overview map, carbohydrate metabolism, amino acid metabolism and glycan biosynthesis and metabolism. 

It has been reported that genomic profile is substantially different between MCF-7 and MDA-MB-231 cells. In the present study, we found that only 41 and 22 KEGG pathways were annotated in MCF-7 and MDA-MB-231 cells. It is worth noting that different cell lines carried specific genomic alternations, possibly due to different response to chlorophyllides composites. 

### 3.4. Analysis of Common KEGG Pathways

**Figure 4:**
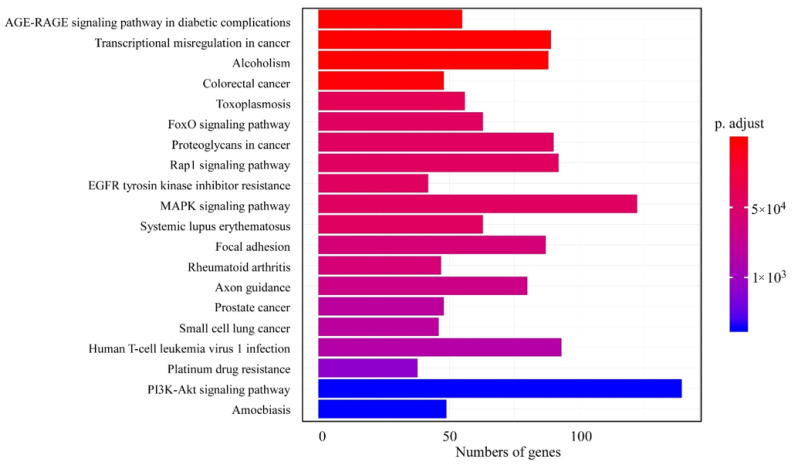
Common KEGG pathways in chlorophyllides composites-treated MCF-7 and MDA-MB-231 cells. The *y*-axis is classification of KEGG, and the *x*-axis is the corresponding number of genes.

To further identify the chlorophyllides composites relevant KEGG pathways that were common in both breast cancer cell lines, we compared the KEGG pathways that were enriched in chlorophyllides composites-treated MCF-7 and MDA-MB-231 cells (Figure 4). The top 20 common KEGG pathways were shown, including the PI3K-Akt signaling pathway, MAPK pathway, Rap1 signaling pathway or human T-cell leukemia virus 1 infection. In this study, we focused more on the underlying pathways that are related to cancer or drug resistance. Hence, the differential expression involved in cancer or drug resistance was compared between MCF-7 and MDA-MB-231 cells (Table 2 and Table 3). The results revealed that 23 and 4 significantly enriched KEGG pathways related to cancer and drug resistance were identified, respectively. We observed that 90, 89 and 74 DEGs were mapped to “proteoglycans in cancer” (hsa05205), “transcriptional misregulation in cancer” (hsa05202) and “viral carcinogenesis” (hsa05203) pathways (Table 2). For the chlorophyllides composites relevant pathways in drug resistance, there were 15, 38, 42 and 45 DEGs related to “antifolate resistance” (hsa01523), “platinum drug resistance” (hsa01524), “EGFR tyrosine kinase inhibitor resistance” (hsa01521) and “endocrine resistance” (hsa01522) pathways (Table 3). 

Li et al. identified the key genes and pathways associated with metastasis of MCF-7 and MDA-MB-231 cells [50]. Further, they identified survival-correlated genes (ALOX15, COL4A6, LMB13, MTAP, PLA2G4A, TAT) and metastasis-associated genes (SNRPN, ARNT2, HDGFRP3, ERO1LB, ERLIN2, YBX2, EBF4). They also identified signaling pathways; metabolic pathways, phagosome pathway, PI3K-AKT signaling pathway, focal adhesion, ECM-receptor interaction, pancreatic secretion and human papillomavirus infection were mainly associated with metastasis. In addition, Sun et al. screened and identified common and specific genes in breast cancer subtypes basal-like, Her2, LumA, LumB and normal-like molecular subtypes [51]. The authors identified 4 common and 34 specific DEGs in different subtypes. Similar to these studies, chlorophyllides composites treatment also affected signal transduction or metabolic progress, indicating that chlorophyllides composites may act on the genes that correlated with metastasis.

### 3.5. Validation of RNA Expression by RT-qPCR

**Figure 5:**
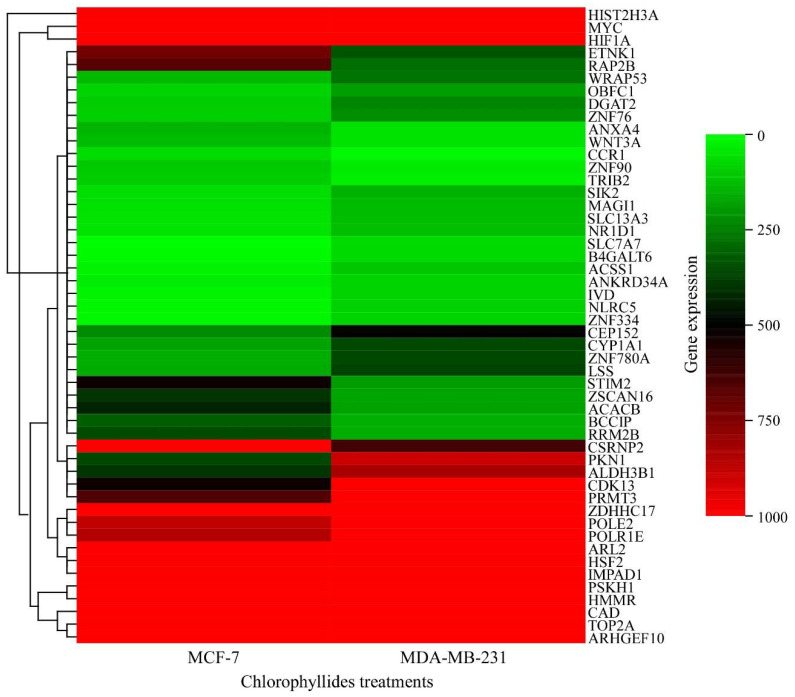
Identification of common DEGs between chlorophyllides composites-treated MCF-7 and MDA-MB-231 cells. Heat map showing the hierarchical cluster of differential expression levels between chlorophyllides composites-treated MCF-7 and MDA-MB-231 cells. The top 50 candidate genes were selected from chlorophyllide-treated MCF-7 and MDA-MB-231 cells. The color scale represents expression values; red indicates the high expression level, and the green refers to the low expression level.

The functions of upregulated and downregulated genes are correlated with chlorophyllides composites treatment. For further understanding the role of chlorophyllides composites in breast cancers and to find potential targets for chlorophyllides composites treatment, gene expression profiles between chlorophyllides composites-treated MCF-7 cells and MDA-MB-231 cells were compared. We analyzed the microarray data sets of upregulated and downregulated genes in MCF-7 and MDA-MB-231 cells to identify specific effects of chlorophyllides composites-induced cytotoxicity. Hierarchical clustering indicated that the DEGs were detected by chlorophyllides composites treatment between MCF-7 and MDA-MB-231 cells (Figure 5). Collectively, the levels of 52 genes were differentially expressed in MCF-7 and MDA-MB-231 cells. The expression of 16 genes was upregulated in MCF-7 cells, such as annexin A4 (ANXA4), chemokine C-C motif receptor 1 (CCR1), stromal interaction molecule 2 (STIM2), ethanolamine kinase 1 (ETNK1) and member of RAS oncogene family (RAP2B). There were 36 upregulated expressed genes in MDA-MB-231 cells, such as membrane-associated guanylate kinase WW and PDZ domain containing 1 (MAGI1), NLR family CARD domain containing 5 (NLRC5), solute carrier family 7 membrane 7 (SLC7A7), protein kinase N1 (PKN1) and DNA topoisomerase II alpha 170 kDa (TOP2A). The log2 FC of CCR1, STIM2, ETNK1 and RAP2B from microarray resulted in chlorophyllides composites-treated MCF-7 cells that were 5.954, 2.783, 2.181 and 2.375, respectively. The log2 FC of MAGl1, NLRC5, SLC7A7, TOP2A and PKN1 from microarray results in chlorophyllides-treated MDA-MB-231 cells were 2.307, 5.824, 22.208, 2.52 and 2.32, respectively. 

**Figure 6:**
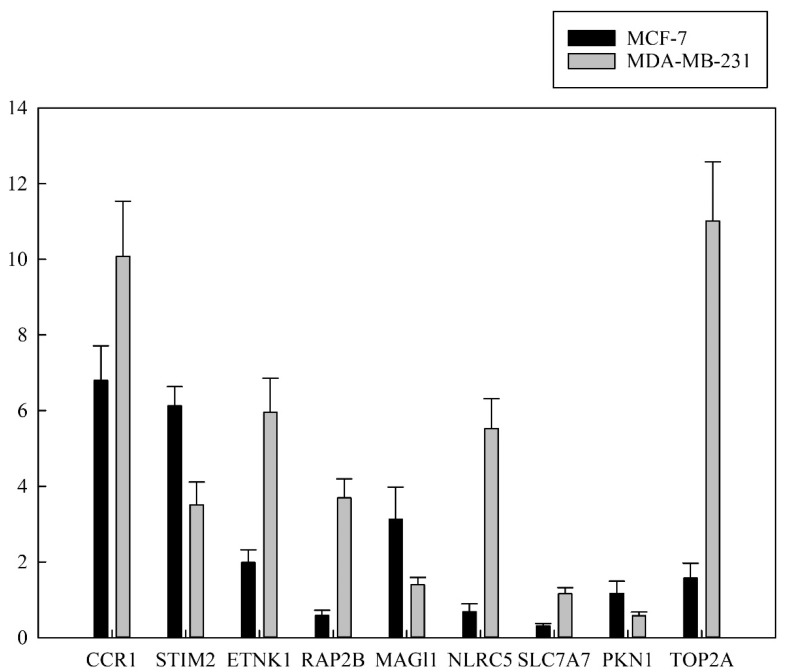
Validation of relative expression levels from RNA-seq and real-time polymerase chain reaction. Nine randomly selected differentially expressed genes (DEGs) were validated for the expression level from chlorophyllide-treated MCF-7 and MDA-MB-231 cells. Expression of target genes was normalized to GAPDH as a reference gene, and statistically significant differences from control are presented, with *p* < 0.05. The *x*-axis denotes nine genes. The *y*-axis refers to the relative expression level with the mean ± standard deviation of five replicates.

To validate the expression level of CCR1, STIM2, ETNK1, RAP2B, MAGl1, NLRC5, SLC7A7, TOP2A and PKN1 identified in chlorophyllides composites-treated MCF-7 and MDA-MB-231 cells, we performed RT-qPCR to evaluate their expression levels of genes as mentioned above (Figure 6). Nine randomly selected genes were detected in the chlorophyllides composites-treated MCF-7 and MDA-MB-231 cells by RT-qPCR, and the primers were listed in Table 1. The FC of CCR1, STIM2, ETNK1, MAGl1 and TOP2A by RT-qPCR were 6.798, 2.687, 5.75 and 1.574, respectively. The FC of above-mentioned genes were consistent with the expression changes detected by microarray dataset. In MDA-MB-231 cells, the expression level of CCR1, STIM2, ETNK1, RAP2B, MAGl1, NLRC5, SLC7A7 and TOP2A were significantly upregulated, while PKN1 was significantly downregulated. The FC of CCR1, STIM2, ETNK1, RAP2B, NLRC5, SLC7A7 and TOP2A were 10.07, 3.508, 5.95, 3.694, 5.523, 11.015 and 1.159, respectively. The FC of STIM2, RAP2B and NLRC5 were similar to the expression changes detected by RT-qPCR and microarray dataset. 

The genes mentioned below were identified and mapped to the KEGG database, and their association was observed (Table 4). It has been reported that the natural products from plant sources have various biological activities, such as anti-oxidation, anti-inflammation or anti-proliferation [5]. Many of the reported anti-cancer effects of natural compounds also target cellular proteins that play important roles in signal transduction, apoptosis or cell cycle arrest [52]. By the bioinformatics analysis, we found that many DEGs were enriched in signaling and cellular process, such as CCR1, STIM2, ETNK1, RAP2B, MAGI1, NLRC5, SLC7A7, PKN1 and TOP2A. Previously, we have demonstrated that purified chlorophyllides composites could be a potential candidate for combination therapy to breast cancers with multiple drug resistance [38]. Therefore, we focused on several interesting factors that were differentially affected by chlorophyllides composites treatment in MCF-7 and MDA-MB-231 cells. CCR1 expression was upregulated in both MCF-7 and MDA-MB-231 cells. CCR1 belonged to the G protein-linked receptor superfamily. The expression of CCR1 has been reported on tumor cells, peripheral blood cells, immune cells and stromal cells. After the binding of CC chemokine (e.g., CCL14, CCL15, CCL16), CCR1 exhibited important roles in tumor invasion and metastasis in several cancers [53,54,55,56,57]. STIM2 was upregulated in both MCF-7 and MDA-MB-231 cells, and the FC in both cells was in accordance with RT-qPCR and microarray data. STIM2 is an endoplasmic reticulum-associated Ca^2+^-sensing protein that responded to endoplasmic reticulum Ca^2+^ store depletion and transduced this cellular signal to Orai1 channel proteins [58,59]. Previous studies have demonstrated that disturbance of STIM is associated with the pathogenesis of several diseases, such as autoimmune disorders, cancer, cardiovascular disease, ageing, Alzheimer’s and Huntington’s diseases [60,61,62]. ETNK1 expression was upregulated in both MCF-7 and MDA-MB-231 cells. ETNK1 is an ethanolamine-specific kinase that catalyzes the phosphorylation of ethanolamine to generate phosphoethanolamine, which is the first step for biosynthesis of phosphatidylethanolamine. Previous studies have reported that mutations of ETNK1 may play important roles in oncogenesis [63,64]. RAP2B is a member of the Ras oncogene family that was upregulated by chlorophyllides composites in MDA-MB-231 cells. As signaling effectors of GTPase-binding protein Rap, Rap2B mediated various biological functions, including regulating the p53-mediated pro-survival function, and binding phospholipase C-*ε* and interferon-*γ* that promote the development of tumors [65]. 

The increased levels of MAGI1 by chlorophyllides composites was observed in the MCF-7 and MDA-MB-231 cells. MAGI1 is an important protein that is transmitted from extracellular environment to intracellular signaling pathways [66,67]. It has been reported that MAGI1 functioned as a tumor suppressor in several tumors (e.g., cervical cancer, leukemia, colorectal cancer, hepatocellular carcinoma, and gastric cancer. The expression of the NLRC5 gene was upregulated in MDA-MB-231 cells, and the relative expression between MCF-7 and MDA-MB-231 cells was strongly downregulated by more than 5.523-fold. This finding is similar to the expression levels observed in microarray data (5.824 fold). NLRC5 belongs to a large protein family that is involved in the regulation of inflammatory response in tumors. Functions of NLRC5 in tumors remain as a debate [68]. NLRC5 overexpression upregulated the MHC class I-mediated antigen presentation pathway that leads to immune escape of tumor cells. In contrast, new evidence has demonstrated that NLRC5 could promote tumor malignancy [69]. Similarly, the expression of the SLC7A7 gene was upregulated in MDA-MB-231 cells, and the relative expression between MCF-7 and MDA-MB-231 cells was strongly downregulated as observed by more than 35.4-fold. The *SLC7A7* gene encodes for the y^+^LAT1 transporter [70,71]. The y^+^LAT1 transporter was responsible for exchanging cationic amino acids with neutral amino acids and sodium at epithelial cells of the kidney and intestine. It has been reported that mutation in *SLC7A7* caused a rare inherited metabolic disorder of dibasic amino acid transport-lysinuric protein intolerance. 

The expression levels of the PKN1 gene in MCF-7 cells were higher in comparison to MDA-MB-231 cells. PKN1 is a member of the protein kinase C superfamily. Disruption of PKN1 kinase activity is involved in several human diseases, including cancer. A previous study has demonstrated that mitotic phosphorylation is essential for PKN1’s oncogenic function [72]. It was reported that PKN1 acted as a RhoA effector that transduced androgen-responsiveness to serum response factor [73]. Overexpression of PKN1 occurred during clinical castration-recurrent prostate cancer progression, stimulated tumor growth and shortened the survival of prostate cancer xenograft. Since PKN1 belongs to the kinase family, which function as signaling proteins and are identified as successful targets for cancer treatment, it is reasonable to suggest that chlorophyllides composites may interact with PKN1 or inhibit the activity of PKN1. 

TOP2 A was upregulated in MCF-7 and MDA-MB-231 cells. TOP2A acted as a DNA replication- and cell division-regulating enzyme. The overexpression of TOP2A was reported in several human cancers, including breast cancer and hepatocellular carcinoma [74]. Also, high levels of expression of chromatin regulatory genes (e.g., TOP2A) increased DNA accessibility and then led to greater anthracycline benefit [75]. Amplification of the *MYC* oncogene was the most common abnormality in breast cancer cells, which is similar to the previous study [50]. We also observed that *Myc* oncogenes were upregulated in chlorophyllides composites-treated MDA-MB-231 cells. 

Breast cancer is regarded as a heterogeneous disease characterized by molecular aberrations and varying histologic and biological features. Microarray-based gene expression profiling had a significant impact on the understanding of breast cancers. The molecular classification system and prognostic multigene classifiers by microarrays was developed [10]. For example, correlation was found between immunohistochemistry and gene profiling of breast cancer, especially basal-like breast carcinoma. The intrinsic 40-gene set was found to classify breast cancer subtype and genes expression differentiations by microarray [76]. In addition, the gene expression profiling from triple negative breast cancer (TNBC) patients by next generation sequencing assay targeted all coding regions of 229 common cancer-related genes [77]. Genetic alterations in TNBC by next-generation sequencing assays was successfully detected [78]. Nonetheless, the functional annotation of common or specific unigenes provided ample numbers of candidate genes and valuable information about biological features of chlorophyllides composites treatment in this study. 

## 4. Conclusions

Chlorophyllides composites could be mass manufactured from chlorophyll using chlorophyllase. In addition, chlorophyllides composites clearly exhibited amazing anticancer activities to breast cancer cell lines (MCF-7 or MDA-MB-231) [37]. To the best of our knowledge, this study is the first to evaluate the effects of chlorophyllides composites on MCF-7 and MDA-MB-231 cells by microarray profile. Moreover, it is also first to identify the global gene expression pattern from the chlorophyllides-treated group. Results indicated that 124 and 77 differentially expressed genes in MCF-7 cells and MDA-MB-231 cells after chlorophyllides composites treatment (A ≥ 2-fold change) was evident. Among these, it is possible to highlight that the expression of CCR1, STIM2, ETNK1, MAGl1 and TOP2A were upregulated in both chlorophyllides composites treated-MCF-7 and MDA-MB-231 cells, indicating that chlorophyllides composites may specifically target or inhibit the activity of these genes. Altogether, these results provide valuable information on the molecular mechanisms induced by chlorophyllides composites in MCF-7 and MDA-MB-231 cells. The DEGs of NLRC5, SLC7A7 and PKN1 may be used as therapy targets that facilitate the development of botanical drugs. 

## Figures and Tables

**Table 1:** Primer name, sequence, target gene and product size used in the present study.

Name	Sequence	Target Gene	Product Size (bp)
GAPDH-F	ATCACTGCCACCCAGA AGAC	*GAPDH*	460
GAPDH-R	ATGAGGTCCACCACCCTGTT		
CCR1-F	AGAAGCCGGGATGGAAACTC	*CCR1*	165
CCR1-R	TTCCAACCAGGCCAATGACA		
STIM2-F	AGTCTTTGGGACTCTGCACG	*STIM2*	129
STIM2-R	TGTTGCCAGCGAAAAAGTCG		
ETNK1-F	CCAAAGCATGTCTGCAACCC	*ETNK1*	114
ETNK1-R	AAGCAGAAGCCTTGACCCTC		
RAP2B-F	AGCTTCCAGGACATCAAGCC	*RAP2B*	190
RAP2B-R	AGGCTTTGTTTTTGGCCGAC		
MAGIL-F	GCCTTGCACAACCCGATCT	*MAGIL*	150
MAGIL-R	GGCTTGGGTGTCCCATAATAG		
NLRC5-F	ACCTTAAGCCTGTGTCCACG	*NLRC5*	115
NLRC5-R	CTGTGAACCTGCCACAGCA		
SLC7A7-F	CTCACTGCTTAACGGCGTGT	*SLC7A7*	170
SLC7A7-R	CCAGTTCCGCATAACAAAGG		
PKN1-F	GCCATCAAGGCTCTGAAGAA	*PKN1*	136
PKN1-R	GTCTGGAAACAGCCGAAGAG		
TOP2A-F	CTTTGGCTCGATTGTTATTTCC	*TOP2A*	142
TOP2A-R	CCCAGTACCGATTCCTTCAG		

**Table 2:** Analysis of chlorophyllides composites relevant pathway in cancers.

Pathway ID	Pathway Description	Number of DEGs			All Genes with Pathway Annotation	*q*-Value
		Up	Down	Total DEGs		
hsa05202	Transcriptional misregulation in cancer	47	42	89 (3.749%)	186 (2.347%)	1.296 × 10^−5^
hsa05203	Viral carcinogenesis	34	40	74 (3.117%)	201 (2.536%)	0.0428029
hsa05205	Proteoglycans in cancer	35	55	90 (3.791%)	204 (2.574%)	0.0002796
hsa05210	Colorectal cancer	22	26	48 (2.022%)	86 (1.085%)	2.367 × 10^−5^
hsa05211	Renal cell carcinoma	17	12	29 (1.222%)	69 (0.871%)	0.0433838
hsa05212	Pancreatic cancer	15	22	37 (1.559%)	69 (0.871%)	0.0021288
hsa05213	Endometrial cancer	13	14	27 (1.137%)	69 (0.871%)	0.0159683
hsa05214	Glioma	17	17	34 (1.432%)	69 (0.871%)	0.0118167
hsa05215	Prostate cancer	25	23	48 (2.022%)	97 (1.224%)	0.0005016
hsa05216	Thyroid cancer	10	11	21 (0.885%)	37 (0.467%)	0.0032991
hsa05217	Basal cell carcinoma	9	15	24 (1.011%)	63 (0.795%)	0.1293504
hsa05218	Melanoma	15	16	31 (1.306%)	72 (0.909%)	0.0298569
hsa05219	Bladder cancer	9	9	18 (0.758%)	41 (0.517%)	0.0669118
hsa05220	Chronic myeloid leukemia	14	22	36 (1.516%)	76 (0.959%)	0.0045089
hsa05221	Acute myeloid leukemia	15	16	31 (1.306%)	67 (0.845%)	0.0118167
hsa05222	Small cell lung cancer	13	33	46 (1.938%)	92 (1.161%)	0.0005016
hsa05223	Non-small cell lung cancer	13	20	33 (1.390%)	66 (0.833%)	0.0029084
hsa05224	Breast cancer	34	27	61 (2.570%)	147 (1.855%)	0.0066776
hsa05225	Hepatocellular carcinoma	32	40	72 (3.033%)	168 (2.120%)	0.0016521
hsa05226	Gastric cancer	30	28	58 (2.443%)	149 (1.880%)	0.0283904
hsa05230	Central carbon metabolism in cancer	13	17	30 (1.264%)	69 (0.871%)	0.0286728
hsa05231	Choline metabolism in cancer	20	16	36 (1.516%)	98 (1.237%)	0.1137391
hsa05235	PD-L1 expression and PD-1 checkpoint pathway in cancer	14	21	35 (1.474%)	89 (1.123%)	0.0624114

**Table 3:** Drug resistance pathway analysis of chlorophyllides composites.

Pathway ID	Pathway Description	Number of DEGs			All Genes with Pathway Annotation	*q*-Value
		Up	Down	Total DEGs		
hsa01521	EGFR tyrosine kinase inhibitor resistance	22	20	42 (1.769%)	79 (0.997%)	0.0002796
hsa01522	Endocrine resistance	24	21	45 (1.896%)	98 (1.237%)	0.0032168
hsa01523	Antifolate resistance	5	10	15 (0.632%)	31 (0.391%)	0.0464471
hsa01524	Platinum drug resistance	15	23	38 (1.601%)	73 (0.921%)	0.0006735

**Table 4:** Differentially expressed genes (DEGs) regulate after chlorophyllides treatment between MCF-7 and MDA-MB-231 cells.

Description	Gene Name	Log2 FC *	KEGG Pathway
Up regulation (MCF-7-chlorophyllides/MDA-MB-231-chlorophyllides)			
annexin A4	*ANXA4*	1.3495564	hsa04974
C-C motif chemokine receptor 1	*CCR1*	2.573958	ko04060, ko04061, ko04062, ko05163, ko05167
stromal interaction molecule 2	*STIM2*	1.4764014	hsa04020
ethanolamine kinase 1	*ETNK1*	1.1246655	hsa00564, hsa01100
RAP2B, member of RAS oncogene family	*RAP2B*	1.2477774	NA
BRCA2 and CDKN1A interacting protein	*BCCIP*	1.0360939	NA
ribonucleotide reductase M2 B	*RRM2B*	1.1502474	hsa00230, hsa00240, hsa00480, hsa00983, hsa01100, hsa04115
cysteine-serine-rich nuclear protein 2	*CSRNP2*	1.155312	NA
serine kinase H1	*PSKH1*	1.1608988	NA
zinc finger and SCAN domain containing 16	*ZSCAN16*	1.175633	NA
histone cluster 2, H3a	*HIST2H3A*	1.2060455	hsa04613, hsa05034, hsa05131, hsa05202, hsa05322
wingless-type MMTV integration site family, member 3A	*WNT3A*	1.2382799	hsa04150, hsa04310, hsa04390, hsa04550, hsa04916, hsa04934, hsa05010, hsa05022, hsa05165, hsa05200, hsa05205, hsa05206, hsa05217, hsa05224, hsa05225, hsa05226
acetyl-CoA carboxylase beta	*ACACB*	1.2477973	hsa00061, hsa00620, hsa00640, hsa01100, hsa04152, hsa04910, hsa04920, hsa04922, hsa04931
zinc finger protein 90	*ZNF90*	1.4318171	hsa05168
hyaluronan-mediated motility receptor	*HMMR*	1.4742341	ko04512
tribbles pseudokinase 2	*TRIB2*	1.5311222	NA
Down- regulation (MCF-7-chlorophyllides/MDA-MB-231-chlorophyllides)			
membrane associated guanylate kinase, WW and PDZ domain containing 1	*MAGI1*	−1.2064317	hsa04015, hsa04151, hsa04530, hsa05165
NLR family, CARD domain containing 5	*NLRC5*	−2.5420052	NA
solute carrier family 7 (amino acid transporter light chain, y+L system), member 7	*SLC7A7*	−4.4729806	hsa04974
protein kinase N1	*PKN1*	−1.3322328	hsa04151, hsa04621, hsa05132, hsa05135
topoisomerase (DNA) II alpha 170kDa	*TOP2A*	−1.1590858	hsa01524
UDP-Gal:betaGlcNAc beta 1,4- galactosyltransferase, polypeptide 6	*B4GALT6*	−3.2967566	ko00600, ko01100
zinc finger protein 334	*ZNF334*	−2.4680017	hsa05168
acyl-CoA synthetase short-chain family member 1	*ACSS1*	−2.1925126	hsa00010, hsa00620, hsa00630, hsa00640, hsa01100, hsa01200
isovaleryl-CoA dehydrogenase	*IVD*	−1.8816549	ko00280, ko01100
ADP-ribosylation factor-like 2	*ARL2*	−1.8091316	NA
Rho guanine nucleotide exchange factor 10	*ARHGEF10*	−1.9429478	ko04270, ko04611, ko04810, ko04928, ko05130, ko05135, ko05163, ko05200, ko05205, ko05417
cyclin-dependent kinase 13	*CDK13*	−1.3298924	NA
diacylglycerol O-acyltransferase 2	*DGAT2*	−1.3269336	ko00561, ko01100, ko04975
solute carrier family 13 (sodium-dependent dicarboxylate transporter), member 3	*SLC13A3*	−1.3249987	NA
nuclear receptor subfamily 1, group D, member 1	*NR1D1*	−1.2920206	ko04710
zinc finger protein 76	*ZNF76*	−1.2438793	hsa05168
ankyrin repeat domain 34A	*ANKRD34A*	−1.2280619	NA
salt-inducible kinase 2	*SIK2*	−1.2129109	ko04922
v-myc avian myelocytomatosis viral oncogene homolog	*MYC*	−1.2045108	ko04010, ko04012, ko04110, ko04151, ko04218, ko04310, ko04350, ko04390, ko04391, ko04550, ko04630, ko04919, ko05132, ko05160, ko05161, ko05163, ko05166, ko05167, ko05169, ko05200, ko05202, ko05205, ko05206, ko05207, ko05210, ko05213, ko05216, ko05219, ko05220, ko05221, ko05222, ko05224, ko05225, ko05226, ko05230
zinc finger protein 780A	*ZNF780A*	−1.1839843	hsa05168
oligonucleotide/oligosaccharide-binding fold containing 1	*OBFC1*	−1.1801386	NA
lanosterol synthase	*LSS*	−1.163355	ko00100, ko01100, ko01110, ko01130
zinc finger, DHHC-type containing 17	*ZDHHC17*	−1.1437738	NA
carbamoyl-phosphate synthetase 2, aspartate transcarbamylase, and dihydroorotase	*CAD*	−1.1388409	hsa00240, hsa00250, hsa01100, hsa01240
centrosomal protein 152kDa	*CEP152*	−1.1385807	NA
hypoxia inducible factor 1, alpha subunit	*HIF1A*	−1.0661852	ko04066, ko04137, ko04140, ko04212, ko04361 Axon regeneration ko04659, ko04919, ko05167, ko05200, ko05205, ko05211, ko05230, ko05231, ko05235
aldehyde dehydrogenase 3 family, member B1	*ALDH3B1*	−1.063386	hsa00010, hsa00340, hsa00350, hsa00360, hsa00410, hsa00980, hsa00982, hsa01100
polymerase (DNA directed), epsilon 2, accessory subunit	*POLE2*	−1.0376518	ko03030, ko03410, ko03420
arginine methyltransferase 3	*PRMT3*	−1.0357205	NA
polymerase (RNA) I polypeptide E, 53kDa	*POLR1E*	−1.0155994	ko03020
cytochrome P450, family 1, subfamily A, polypeptide 1	*CYP1A1*	−1.0129887	ko00140, ko00380, ko00830, ko00980, ko01100, ko04913, ko05204
WD repeat containing, antisense to TP53	*WRAP53*	−1.0114268	NA
heat shock transcription factor 2	*HSF2*	−1.0091325	ko03000
inositol monophosphatase domain containing 1	*IMPAD1*	−1.0071035	ko00562, ko00920, ko01100, ko01120, ko01130, ko04070

NA: not available; Log2 FC *: Log2 FC (MCF-7-chlorophyllides/MDA-MB-231-chlorophyllides).

## Data Availability

Data is contained within the article or supplementary material.

## Supplementary Materials

The following are available online at https://www.mdpi.com/article/10.3390/molecules27123950/s1, Figure S1: HPLC analysis profiles of chlorophyllides composites [37,38].

## Sample Availability

Samples of the compounds-chlorophyllides conpositesare available from the author (Jei-Fu Shaw).

## Abbreviations

(ANXA4)	annexin A4
(BP)	biological process
(CC)	cellular component
(CCR1)	chemokine C-C motif receptor 1
(TOP2A)	DNA topoisomerase II alpha 170 kDa
(ER)	estrogen receptor
(ETNK1)	ethanolamine kinase 1
(FC)	fold change
(HER2)	human epidermal growth receptor 2
(RAP2B)	member of RAS oncogene family
(MAGI1)	membrane associated guanylate kinase WW and PDZ domain containing 1
(MF)	molecular function
(NLRC5)	NLR family CARD domain containing 5
(SLC7A7)	solute carrier family 7 membrane 7
(STIM2)	stromal interaction molecule 2
(PR)	progesterone receptor
(PKN1)	protein kinase N1
(TNBC)	triple negative breast cancer
(RT-qPCR)	quantitative reverse transcription PCR
