# Supplementary material for: Screening of Specific and Common Pathways in Breast Cancer Cell Lines MCF-7 and MDA-MB-231 Treated with Chlorophyllides Composites
Source: Molecules. 2022 Jun 20;27(12):3950. doi: 10.3390/molecules27123950 (PMC9229827; doi:10.3390/molecules27123950)
Supplement: Supplementary file 1 [file molecules-27-03950-s001.zip › molecules-1739944-supplementary.pdf]

Screening of Specific and Common pathways in Breast Cancer Cell Lines MCF-7 and MDA-MB-231 Treated with Chlorophyllides Composites

Supplementary

Figure S1

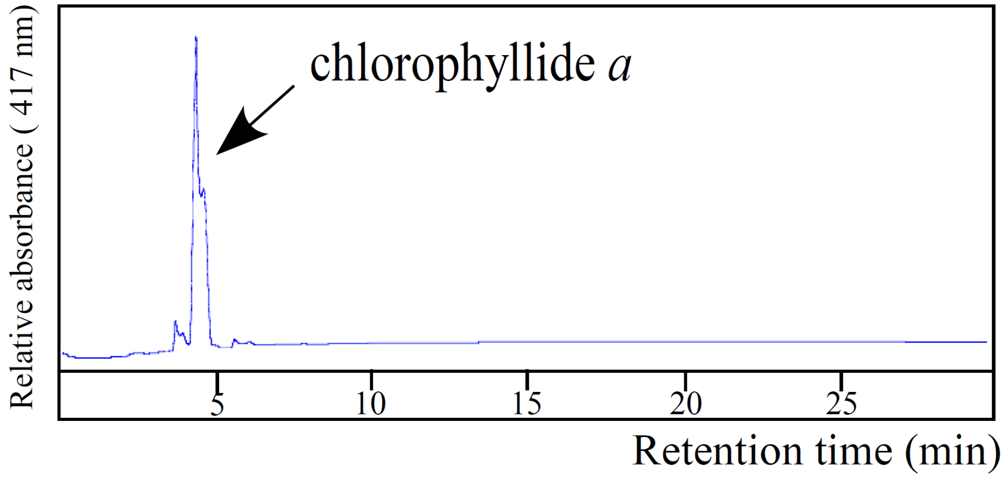

Figure S1. HPLC analysis profiles of chlorophyllides composites. The chlorophyllides composites were separated by HPLC and detected at 417 nm from 0 to 80 min. Chlorophyllide *a* was detected within 5 min. Chlorophyllide *a* are > 95% pure by HPLC analysis. The HPLC traces is similar to that from our previous studies[37,38].
